# Deterministic patterns in single-cell transcriptomic data

**DOI:** 10.1038/s41540-025-00490-5

**Published:** 2025-01-11

**Authors:** Zhixing Cao, Yiling Wang, Ramon Grima

**Affiliations:** 1 State Key Laboratory of Bioreactor Engineering, East China University of Science and Technology, Shanghai, China; 2 Department of Chemical Engineering, Queen’s University, Kingston, ON Canada; 3 School of Biological Sciences, University of Edinburgh, Edinburgh, UK

**Keywords:** Cellular noise, Stochastic modelling, Molecular fluctuations

## Abstract

We report the existence of deterministic patterns in statistical plots of single-cell transcriptomic data. We develop a theory showing that the patterns are neither artifacts introduced by the measurement process nor due to underlying biological mechanisms. Rather they naturally emerge from finite sample size effects. The theory precisely predicts the patterns in data from multiplexed error-robust fluorescence in situ hybridization and five different types of single-cell sequencing platforms.

Gene expression does not occur in a regular manner—rather it occurs in randomly timed bursts of intense transcriptional activity, separated by long pauses^1^. The stochastic nature of gene expression manifests as large cell-to-cell heterogeneity in the number of gene products^2–4^. Variability in the number of mRNA per cell can be quantified by single-cell transcriptomic techniques such as single-cell RNA sequencing (scRNA-seq^5–7^), single-molecule fluorescence in situ hybridization (smFISH^8–10^) and multiplexed error-robust fluorescence in situ hybridization (MERFISH^11–13^). The use of single-cell resolved data is crucial because many heritable variations in gene function are masked when the expression is averaged over a large number of cells^14^. Recently, the fitting of stochastic models of gene expression to single-cell sequencing data has also made it possible to estimate the transcriptional parameters (the burst size and the burst frequency) for each gene^15,16^. Estimation of these parameters provides insight into the relationship between core promoter elements (such as gene length, TATA, and initiator elements) and the mode of transcriptional activity.

The distributions of the number of mRNA per cell for many genes are fit well by a negative binomial distribution^17,18^. The two parameters of this distribution are completely determined by two physically meaningful quantities: the mean transcript count and the Fano factor (a measure of variability defined as the ratio of the variance and the mean of transcript counts^19^). Note that the Fano factor (FF) equals one if the distribution of counts is Poissonian; it is greater (smaller) than 1 if the distribution of counts is wider (narrower) than a Poisson with the same mean. Cases of sub-Poisson expression (FF <1^9^), Poisson expression (FF = 1^20^) and super-Poisson expression (FF >1^21^) have been reported in the literature. It hence follows that a plot of the mean vs Fano factor, where each point is a gene, provides a simple way to visualize gene-to-gene differences across the whole transcriptome. An alternative but equivalent plot shows the burst size vs the mean^1^ where we note that the burst size is obtained by subtracting 1 from the Fano factor (when mRNA is distributed according to a negative binomial). Hitherto, the general properties of mean-Fano factor plots remain unexplored.

In this paper, we calculate mean-FF plots of yeast, mouse, and human genomes using data from six different types of single-cell transcriptomic technologies. Surprisingly, we find that the plots show deterministic patterns, which at first sight appear to be difficult to reconcile with the underlying stochasticity of gene expression data. We formulate a theory showing that independent of the type of transcriptional activity (bursty or constitutive) and of the origin of the noise (intrinsic or extrinsic), intricate deterministic patterns in the mean-Fano factor plots are a natural but non-intuitive consequence of the typically small sample size of high-quality single-cell transcriptomic data. Remarkably, the theory precisely predicts the patterns in the mean-Fano factor plots of all datasets analyzed.

Genome-wide transcript count data can be organized as a matrix where the integer at the *i*th row and *j*th column is the transcript count for gene *i* in cell *j*. To understand how the Fano factor might depend on the mean expression, the type of measurement technique, and the cell types, we computed plots of the mean versus the Fano factor using yeast, mouse, and human data collected using MERFISH and five different types of scRNA-seq protocols. The plots are shown in Fig. 1. Note that each point in this plot represents a gene, and we have specifically focused on the region where the mean count is less than 1.

**Fig. 1:**
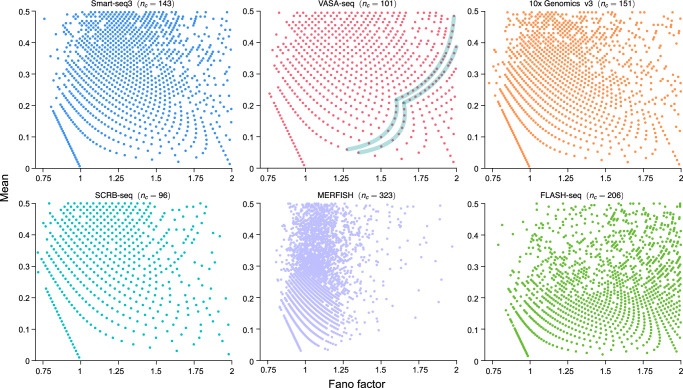
Mean-Fano factor plots of six genomic datasets using various types of scRNA-seq protocols and MERFISH. Each plot shows a deterministic pattern; one pattern is highlighted for VASA-seq data. Each point represents the mean and Fano factor computed from the transcript counts of a gene in a finite number *n*_*c*_ of cells (some genes may have the same coordinates). Information on each dataset can be found in Supplementary Note 1.

Contrary to our expectations, the plots are not particularly different from each other and in all cases they show the existence of deterministic patterns. This is particularly surprising given the stochastic nature of gene expression^2^ and the large differences between sequencing protocols^22^. Altogether, the evidence suggests that the patterns are not an artifact of the experimental technique and that they may have an underlying common origin due to fundamental mathematical, physical, chemical, or biological principles.

An important observation is that the points in the mean-Fano factor plot appear to fall on a set of monotonically decreasing curves from left to right. In Fig. 2a, we highlight three of these curves in the plot generated using the 10x Genomics v3 platform and refer to them as Curves 0, 1, and 2. At first glance, it appears that the vertical distance between two successive points on the same curve, Δ*y*, almost always takes the same value (inset of Fig. 2a). This quantization is verified in Fig. 2b, where we show that for all points on Curves 0, 1 and 2 in the six datasets in Fig. 1, Δ*y* is a multiple of 1/*n*_*c*_ where *n*_*c*_ is the number of cells in each dataset (number of columns in the count matrix). Furthermore, in 80% of the cases, Δ*y* = 1/*n*_*c*_. The 20% of the cases where Δ*y* is a multiple of 1/*n*_*c*_ originates from missing data points (either the gene corresponding to this point does not exist or was not detected due to the imperfect capture of a proportion of transcripts). For example, let us say the missing point is the *i*th point (measured by the vertical distance of a point to the *x* axis). Then it follows that the distance between two successive points, the *i* − 1th point and the *i* + 1th point, is 2/*n*_*c*_.

**Fig. 2:**
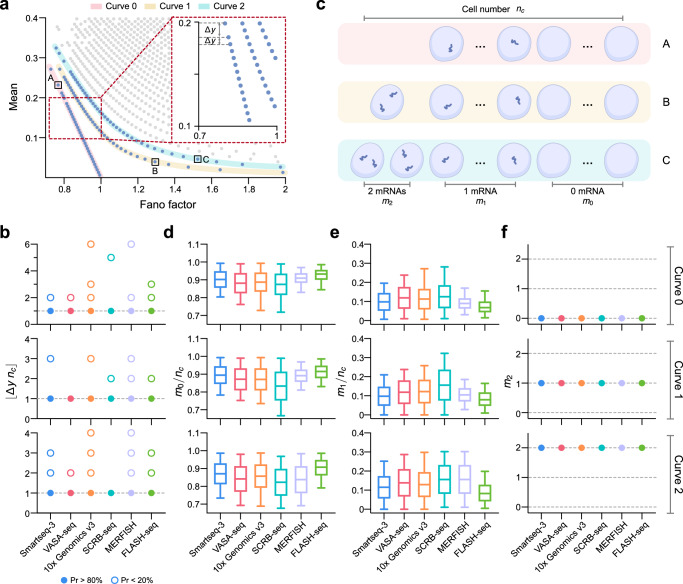
Analysis of the patterns in the mean-Fano factor plot of single-cell transcriptomic data. **a** Points in the mean-Fano factor plot generated using the 10x Genomics v3 technology are arranged on distinct curves. We highlight three of them and call them Curves 0, 1 and 2. The inset zooms in on the curves and shows the existence of a periodic distance between the points on the same curve. The vertical distance between two successive points on the same curve is denoted by Δ*y*. **b** For all six datasets in Fig. 1, Δ*y* (computed from Curves 0–2) is a multiple of 1/*n*_*c*_ where *n*_*c*_ is the number of cells used to compute the mean and the Fano factor of transcript number fluctuations. Note that in 80% of cases, Δ*y* = 1/*n*_*c*_ (solid circles). **c** Variation in the number of transcripts per cell for three genes A, B, and C on Curves 0, 1, and 2, respectively, in (**a**). For gene A, cells only have 0 or 1 transcript. For gene B, cells have 0–2 transcripts but only one cell has 2 transcripts. For gene C, cells have 0–2 transcripts but only two cells have 2 transcripts. Note that *m*_*i*_ is the number of cells with exactly *i* transcripts. **d** Fraction of cells with exactly zero transcripts for genes on Curves 0 (top), 1 (middle) and 2 (bottom) for all six datasets. **e** Same as (**d**) but showing fraction of cells with exactly one transcript. Table S1 summarizes the median values in (**d**, **e**). **f** Number of cells with exactly two transcripts for genes on Curves 0 (top), 1 (middle) and 2 (bottom) for all six datasets.

Next, we investigated whether there are any obvious statistical differences between the expression of genes on Curves 0, 1, and 2 in the 10x Genomics v3 dataset. In Fig. 2c, we show that for a gene on Curve 0 (denoted as gene A in Fig. 2a) each cell in the sample has only 0 or 1 transcripts; for a gene on Curve 1 (denoted as gene B in Fig. 2a) one cell has two transcripts and the rest of the cells in the sample have only 0 or 1 transcripts; for a gene on Curve 2 (denoted as gene C in Fig. 2a) two cells have two transcripts and the rest of the cells in the sample have only 0 or 1 transcripts. In Fig. 2d–f, we show that these observations extend to all genes in Curves 0–2 in all six datasets. That is, genes on different curves cannot be distinguished by the fraction of cells with 0 or 1 transcripts, but only by the number of cells which have 2 transcripts. Armed with these observations, we now devise a simple theory that can predict the patterns in the mean-Fano factor plots.

Consider genes sitting on Curve *k* which can have only 0, 1, or 2 transcripts with the proviso that there are only *k* cells in the sample which have exactly two transcripts. Let *n*_*c*_ be the total number of cells. Then the expression data for a gene is given by the vector $\{{n}_{1},{n}_{2},{n}_{3},\cdots \,,{n}_{{n}_{c}-k},2,\cdots \,,2\}$ where *n*_*i*_ is the number of transcripts in cell *i*. It then follows that the mean and the mean squared expression are given by:

1 $$\langle n\rangle =\frac{\mathop{\sum }\nolimits_{i = 1}^{{n}_{c}-k}{n}_{i}+2k}{{n}_{c}}=a+\frac{2k}{{n}_{c}},\quad \langle {n}^{2}\rangle =\frac{\mathop{\sum }\nolimits_{i = 1}^{{n}_{c}-k}{n}_{i}^{2}+{2}^{2}k}{{n}_{c}}=a+\frac{4k}{{n}_{c}},$$

where $\mathop{\sum }\nolimits_{i = 1}^{{n}_{c}-k}{n}_{i}/{n}_{c}=\mathop{\sum }\nolimits_{i = 1}^{{n}_{c}-k}{n}_{i}^{2}/{n}_{c}$ equals some fraction *a*; this is since *n*_*i*_ = 0 or 1 for 1 ≤ *i* ≤ *n*_*c*_ − *k*. Hence the Fano factor is given by

2 $${\rm{FF}}=\frac{\langle {n}^{2}\rangle -{\langle n\rangle }^{2}}{\langle n\rangle }=1-\langle n\rangle +\frac{2k}{{n}_{c}\langle n\rangle },\quad 1\le k\le {n}_{c},$$

where we eliminated the parameter *a* using Eq. (1). Solving for the mean, we finally obtain the equation of Curve *k* in the mean-Fano factor plot:

3 $$\langle n\rangle =1-\,\text{FF}\,,\quad k=0$$

4 $$\langle n\rangle =\frac{1}{2}\left(1-\,{\text{FF}}+\sqrt{\frac{8k+{n}_{c}{{(\text{FF}}-1)}^{2}}{{n}_{c}}}\right),\quad 1\le k\le {n}_{c}.$$

Note that genes on Curve 0 have a Bernoulli distribution of transcript counts because in each cell the count is 0 or 1. Eqs. (3)–(4) explain why the plotting of 10x Genomics data in Fig. 2a shows Curve 0 is a straight line while Curves 1 and 2 have non-zero curvature. It is straightforward to show that for any discrete distribution of transcript numbers, 〈*n*〉 ≥ 1 − FF (Supplementary Note 2). This explains the existence of the empty bottom left triangle bounded from above by Curve 0 in Fig. 2a. In Fig. 3, we verify that the theory is successful in predicting the curves in the mean-Fano factor plots of all six types of single-cell transcriptomic data shown in Fig. 1.

**Fig. 3:**
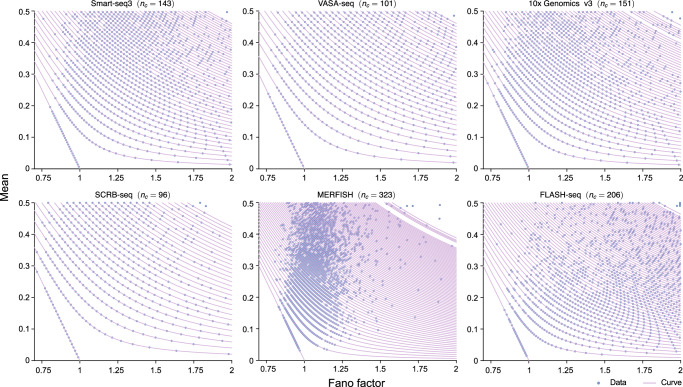
Mean-Fano factor plots of the six sequencing datasets in Fig. 1 and the theoretical predictions given by Eqs. (3)–(4). The curves pass through all points in the dataset thus verifying the accuracy of the theory.

The theory also provides a simple explanation of the quantization along the vertical axis shown in Fig. 2b. Recall that the mean expression 〈*n*〉 of a gene on Curve *k* is given by Eq. (1). Since the numerator of the equation is an integer, it follows that the mean transcript count of the genes on Curve *k* can only differ by an integer multiple of 1/*n*_*c*_. Hence our hypothesis is that by choosing the y-coordinate position of a point to be a multiple of 1/*n*_*c*_ and its x-coordinate to be given by Eq. (2), we can reproduce the full point pattern. In Fig. 4, we verify that this recipe precisely predicts the intriguing pattern in the mean-Fano factor plot of the VASA-seq dataset in Fig. 1. In particular, the position of the data points (shown by the dark blue dots) coincides with the blue open circles predicted by the theory. The success of the simple theory is in large part because its main results agree with those from a more general theory where we consider genes sitting on curves which can have 0, 1, ⋯ , *N* transcripts with the proviso that there are exactly *m*_*i*_ cells in the sample with *i* ≥ 2 transcripts (Supplementary Note 3).

**Fig. 4:**
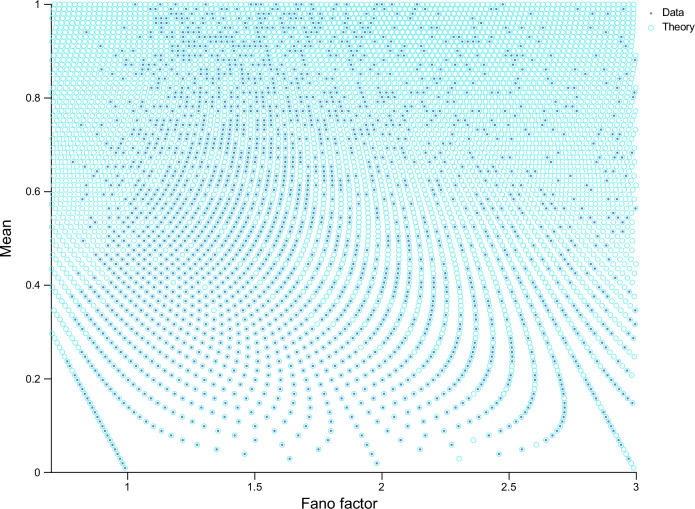
Theory predicts the patterns in the mean-Fano factor plot of VASA-seq data. The dots are calculated from the data (each represents a gene) and the open circles have x-coordinate given by Eq. (2) and y-coordinate given by 〈*n*〉 = *i*/*n*_*c*_ where *n*_*c*_ is the sample size of 101 cells and *i* is a positive integer. Note that some open circles lack corresponding dots due to missing data points. This absence occurs either because the gene corresponding to the point does not exist or it was not detected because typically only a small fraction of the transcriptome of each cell is captured by sequencing methods^22^.

Finally we emphasize that these deterministic patterns do not rely on any mathematical model of gene expression, and the conclusions of our theory hold for any discrete probability distribution that has finite mean and variance. This suggests that the patterns are likely also present in datasets that are not generated by transcriptomic techniques; we verify this prediction in Supplementary Note 4 and Fig. S1.

In this paper, we have reported the existence of patterns in the mean-Fano factor plots summarizing the statistics of data from single-cell transcriptomics. The patterns are only obvious when one zooms in on the region where the mean is small. The emergence of deterministic patterns from highly stochastic data seems counterintuitive, and hence our first hypothesis was that this pattern is due to artifacts introduced by the experimental measurement techniques. However, the similarity of pattern features between five different types of single-cell sequencing data and one single-cell nonsequencing data (MERFISH) suggested that this is not the case. Subsequently, we developed a simple theory that precisely predicts the patterns in all analyzed datasets and shows that they emerge from the typically small sample size (several tens to a few hundreds of cells) of high-quality single-cell transcriptomic data. It is likely that these patterns have not been seen before because often data with a small mean are removed from subsequent data analysis^23^ as a quality control step. This is to avoid issues such as zero-inflation, a phenomenon wherein a higher than anticipated number of zero counts due to non-detection of transcripts are observed across the cell population. However, as technology improves, we expect that the accuracy of single-cell data at very low molecule numbers will increase and may even prove useful in distinguishing cell type. The full biological implications of the deterministic patterns in data from weakly expressed genes remain unclear, which we intend to explore in future studies.

## Supplementary information

Supplementary Information

## Data Availability

The data are uploaded to 10.5281/zenodo.11213863.

## Code availability

The Julia code is deposited in Github.

## References

[1] Sanchez, A. & Golding, I. Genetic determinants and cellular constraints in noisy gene expression. *Science* **342**, 1188–1193 (2013). 24311680 10.1126/science.1242975 PMC4045091

[2] Elowitz, M. B., Levine, A. J., Siggia, E. D. & Swain, P. S. Stochastic gene expression in a single cell. *Science* **297**, 1183–1186 (2002). 12183631 10.1126/science.1070919

[3] Cai, L., Friedman, N. & Xie, X. S. Stochastic protein expression in individual cells at the single molecule level. *Nature* **440**, 358–362 (2006). 16541077 10.1038/nature04599

[4] Taniguchi, Y. et al. Quantifying e. coli proteome and transcriptome with single-molecule sensitivity in single cells. *science* **329**, 533–538 (2010). 20671182 10.1126/science.1188308 PMC2922915

[5] Tang, F. et al. mRNA-seq whole-transcriptome analysis of a single cell. *Nat. Methods* **6**, 377–382 (2009). 19349980 10.1038/nmeth.1315

[6] Zheng, G. X. et al. Massively parallel digital transcriptional profiling of single cells. *Nat. Commun.* **8**, 14049 (2017). 28091601 10.1038/ncomms14049 PMC5241818

[7] Hagemann-Jensen, M. et al. Single-cell RNA counting at allele and isoform resolution using Smart-seq3. *Nat. Biotechnol.* **38**, 708–714 (2020). 32518404 10.1038/s41587-020-0497-0

[8] Wheat, J. C. et al. Single-molecule imaging of transcription dynamics in somatic stem cells. *Nature* **583**, 431–436 (2020). 32581360 10.1038/s41586-020-2432-4 PMC8577313

[9] Weidemann, D. E., Holehouse, J., Singh, A., Grima, R. & Hauf, S. The minimal intrinsic stochasticity of constitutively expressed eukaryotic genes is sub-poissonian. *Sci. Adv.* **9**, eadh5138 (2023). 37556551 10.1126/sciadv.adh5138 PMC10411910

[10] Zhao, L., Fonseca, A., Meschichi, A., Sicard, A. & Rosa, S. Whole-mount smFISH allows combining RNA and protein quantification at cellular and subcellular resolution. *Nat. Plants* **9**, 1094–1102 (2023). 37322128 10.1038/s41477-023-01442-9 PMC10356603

[11] Chen, K. H., Boettiger, A. N., Moffitt, J. R., Wang, S. & Zhuang, X. Spatially resolved, highly multiplexed RNA profiling in single cells. *Science* **348**, aaa6090 (2015). 25858977 10.1126/science.aaa6090 PMC4662681

[12] Xia, C., Fan, J., Emanuel, G., Hao, J. & Zhuang, X. Spatial transcriptome profiling by MERFISH reveals subcellular RNA compartmentalization and cell cycle-dependent gene expression. *Proc. Natl. Acad. Sci. USA* **116**, 19490–19499 (2019). 31501331 10.1073/pnas.1912459116 PMC6765259

[13] Zhang, M. et al. Spatially resolved cell atlas of the mouse primary motor cortex by MERFISH. *Nature* **598**, 137–143 (2021). 34616063 10.1038/s41586-021-03705-x PMC8494645

[14] Wills, Q. F. et al. Single-cell gene expression analysis reveals genetic associations masked in whole-tissue experiments. *Nat. Biotechnol.* **31**, 748–752 (2013). 23873083 10.1038/nbt.2642

[15] Larsson, A. J. et al. Genomic encoding of transcriptional burst kinetics. *Nature* **565**, 251–254 (2019). 30602787 10.1038/s41586-018-0836-1 PMC7610481

[16] Grima, R. & Esmenjaud, P.-M. Quantifying and correcting bias in transcriptional parameter inference from single-cell data. *Biophys. J.* **123**, 4–30 (2024). 37885177 10.1016/j.bpj.2023.10.021 PMC10808030

[17] Grün, D., Kester, L. & Van Oudenaarden, A. Validation of noise models for single-cell transcriptomics. *Nat. methods* **11**, 637–640 (2014). 24747814 10.1038/nmeth.2930

[18] Singer, Z. S. et al. Dynamic heterogeneity and dna methylation in embryonic stem cells. *Mol. Cell* **55**, 319–331 (2014). 25038413 10.1016/j.molcel.2014.06.029 PMC4104113

[19] Kaern, M., Elston, T. C., Blake, W. J. & Collins, J. J. Stochasticity in gene expression: from theories to phenotypes. *Nat. Rev. Genet.* **6**, 451–464 (2005). 15883588 10.1038/nrg1615

[20] Zenklusen, D., Larson, D. R. & Singer, R. H. Single-RNA counting reveals alternative modes of gene expression in yeast. *Nat. Struct. Mol. Biol.* **15**, 1263–1271 (2008). 19011635 10.1038/nsmb.1514 PMC3154325

[21] Dar, R. D. et al. Transcriptional burst frequency and burst size are equally modulated across the human genome. *Proc. Natl. Acad. Sci. USA* **109**, 17454–17459 (2012). 23064634 10.1073/pnas.1213530109 PMC3491463

[22] Ziegenhain, C. et al. Comparative analysis of single-cell RNA sequencing methods. *Mol. Cell* **65**, 631–643 (2017). 28212749 10.1016/j.molcel.2017.01.023

[23] Luecken, M. D. & Theis, F. J. Current best practices in single-cell RNA-seq analysis: a tutorial. *Mol. Syst. Biol.* **15**, e8746 (2019). 31217225 10.15252/msb.20188746 PMC6582955

