# Supplementary material for: Deterministic patterns in single-cell transcriptomic data
Source: NPJ Syst Biol Appl. 2025 Jan 11;11:6. doi: 10.1038/s41540-025-00490-5 (PMC11724867; doi:10.1038/s41540-025-00490-5)
Supplement: Supplementary file 1 — Supplementary Information [file 41540_2025_490_MOESM1_ESM.pdf]

Supplementary Information

Zhixing Cao, Yiling Wang, Ramon Grima

December 30, 2024

Supplementary Note 1

In this section, we describe the collection and preprocessing of count data analyzed in the main text.

- **Smartseq-3:** Unique molecular identifier (UMI) count data (`Smartseq3.HEK.cleanup.UMIcounts.txt`) and the annotation file (`Smartseq3.HEK.cleanup.sample_annotation.txt`) from E-MTAB-8735 provided in Ref. [1] were accessed. Based on the annotation file, 143 HEK293T cells that underwent template switch oligo (TSO) and forward primer addition were specifically chosen to compute the count data matrix.

- **VASA-seq:** The count data for unique fragment identifiers (UFIs) (`GSM5369497_E6.5-1_i1_total.UFICounts.tsv`) were obtained from GSE176588, as described in Ref. [2]. This dataset comprises 101 E6.5 mouse embryo cells.

- **10x Genomics:** The 10x Genomics Chemistry v3 sequencing data was obtained from the file `pbmc_1k_v3_filtered_feature_bc_matrix.h5` on the official 10x Genomics website. Using the Julia command `SparseMatrixCSC`, we processed the sparse matrix into a standard count matrix. The cell annotation file was sourced from `cluster.csv`, located in the `analysis/clustering/graphclust/` path within the `pbmc_1k_v3_analysis.tar` file. For analysis, 151 cells were specifically selected from Cluster 6 of peripheral blood mononuclear cells (PBMCs) derived from a healthy human donor.

- **SCRB-seq:** The data file `41564_2018_330_MOESM3_ESM.xlsx` was obtained from the supplementary materials of Ref. [3]. In this Excel file, `Table_S4` contains the count data, while `Table_S3` provides cell annotation information. From this file, we specifically chose the 96 fission yeast cells processed under the experimental condition "37C_15min".

- **MERFISH:** The count data for U-2 OS cells (human) was obtained from the file `pnas.1912459116.sd12.csv`, as described in Ref. [4]. From this dataset, we analyzed the count data from 323 cells in Batch 3.

- **FLASH-seq:** The count data (`HEK_FS_lowAmplification_250K.rds`) for 206 HEK293T cells was obtained from Mendeley data, as described in Ref. [5]. The count matrix was extracted using the R language (provided as `HEK_FS_lowAmplification_250K.csv`).

Supplementary Note 2 Region with no points in the mean-Fano factor plots

Consider a gene whose counts are described by some arbitrary discrete distribution $P(n)$ with mean $\langle n \rangle$ and variance σ^2. From the definition of the variance of the counts, it follows that

$$\begin{aligned} \sigma^2 &= \langle n^2 \rangle - \langle n \rangle^2 \geq \langle n \rangle - \langle n \rangle^2, \\ \text{FF} &= \frac{\sigma^2}{\langle n \rangle} \geq 1 - \langle n \rangle, \\ \langle n \rangle &\geq 1 - \text{FF}. \end{aligned} \tag{S1}$$

Note that in the first line we have used that $n^2 \geq n$ for any positive integer n. Hence it follows that the region $\langle n \rangle < 1 - \text{FF}$ in the mean-Fano factor plot is devoid of points.

Supplementary Note 3 General theory for patterns in mean-Fano factor plots

Consider genes which can have only 0, 1, 2, $\cdots$, N transcripts per cell with the proviso that there are exactly m_i cells which have exactly i transcripts for $2 \leq i \leq N$. Note that we do not fix the number of cells

with 0 or 1 transcripts, i.e. m_0 and m_1 can vary between different genes. Let the total number of cells be $\sum_{i=0}^{N} m_i = n_c$. It then follows that the mean and the mean squared expression are given by:

$$\langle n \rangle = \frac{\sum_{i=1}^{N} i m_i}{n_c} = a + \frac{\sum_{i=2}^{N} i m_i}{n_c}, \tag{S2}$$

$$\langle n^2 \rangle = \frac{\sum_{i=1}^{N} i^2 m_i}{n_c} = a + \frac{\sum_{i=2}^{N} i^2 m_i}{n_c}, \tag{S3}$$

where m_1/n_c equals some fraction a (it can only take values $0, 1/n_c, \cdots, 1 - \sum_{i=2}^{N} m_i/n_c$). Hence the Fano factor is given by

$$\text{FF} = \frac{\langle n^2 \rangle - \langle n \rangle^2}{\langle n \rangle} = 1 - \langle n \rangle + \frac{\sum_{i=2}^{N} i(i-1) m_i}{n_c \langle n \rangle}, \tag{S4}$$

where we eliminated the parameter a using Eq. (S2). Note that Eqs. (S4) is independent of m_0 and m_1 and hence it is valid independent of the number of cells with 0 or 1 transcripts.

Hence it follows that for a fixed cell number vector $\vec{v} = \{m_2, \cdots, m_N\}$, Eq. (S2) gives the y-coordinate and Eq. (S4) gives the x-coordinate of $1 - \sum_{i=2}^{N} m_i/n_c$ points in the mean-Fano factor plot, where each point corresponds to one or more genes. If we consider all possible values of the entries of the vector $\vec{v}$ and vary N as well then all of the mean-FF plot space is scanned. Since $i(i-1)$ is an even number for $i \geq 2$, it follows from Eq. (S4) that the x-coordinate of a general point is given by Eq. (2) but with the difference that that the curve number k varies between 1 and ∞. The y-coordinates are multiples of $1/n_c$ in the range $1/n_c$ to ∞.

Table S1: Medians of m_0/n_c and m_1/n_c grouped by dataset and theoretical curve number k

	m_0/n_c			m_1/n_c		
	Curve 0	Curve 1	Curve 2	Curve 0	Curve 1	Curve 2
Smartseq-3	0.902	0.895	0.871	0.098	0.098	0.115
VASA-seq	0.881	0.871	0.841	0.119	0.119	0.139
10x Genomics v3	0.907	0.901	0.877	0.093	0.093	0.109
SCRB-seq	0.875	0.844	0.823	0.125	0.146	0.156
MERFISH	0.916	0.898	0.838	0.084	0.099	0.156
FLASH-seq	0.932	0.922	0.910	0.068	0.073	0.080

Supplementary Note 4 Patterns in discrete non-transcriptomic data

To investigate the universality of the predicted deterministic patterns, we analyzed 6 non-transcriptomic data sets, the details of which are described below. The mean-FF plots of these data sets are shown in Fig. S1; here we also show that the theoretical predicted curves (Eqs. (3)-(4) in the main text) perfectly pass through all points in the plot. This observation confirms that the patterns arise universally from the discreteness of count data.

- **Birds in North America:** The data file `PFW_all_2021_2023_June2023_Public.csv` was accessed from Feederwatch. The data collection protocol was reported in Ref. [6] and we focused specifically on observation data from 2022. This data was structured into a count matrix, with each row representing an observation spot and each column representing a bird species.

- **High-energy physics paper citations:** Citation data of high-energy physics papers submitted to arXiv from January 1993 to April 2003 (124 months) were obtained from the files `Cit-HepTh.txt` and `Cit-HepTh-dates.txt` on the Stanford Portal. The data was filtered to focus on 3444 papers submitted from January to December 1995. The number of citations to this group of papers from

papers submitted from January 1996 to December 2002 (84 months) was computed. This citation data was then organized into a count matrix, with each row corresponding to a paper and each column representing a monthly time window.

- **Colorado butterfly:** The data file `Boulder-Abundance.txt` was downloaded from Figshare, the supplementary data of Ref. [7]. The data for the number of butterflies of a certain species observed at a particular observation site was organised into a count matrix, with each column corresponding to a species and each row representing a different site.

- **E-commerce data in UK:** Data was obtained from UCI Machine Learning Repository, as described in Ref. [8]. The data was filtered to focus solely on customer purchases from the United Kingdom. This filtered dataset was then transformed into a standard count matrix, where the value in the i-th row and j-th column represents the number of item i purchased by customer j. Data is provided in the file `e-comm.csv`.

- **Disease outbreak in US:** Weekly data was collected from the website for the Centers for Disease Control and Prevention (CDC) in the United States from 2022 to 2024. Specifically we focused on data from the 10th week of 2024. The data for the number of people with a particular disease in a particular state was organised into a count matrix, with each column corresponding to a state and each row representing an infectious disease or condition. The preprocessed data is stored as `cdc.csv`.

- **LinkedIn job in US:** The employment data was obtained for locations in the USA from the file `postings.csv` on Kaggle. A count matrix was structured with columns representing the 50 states, 1 federal district, and 5 inhabited territories (a total of 56 regions, provided as the file `us-states-territories.csv`) and rows representing different occupations.

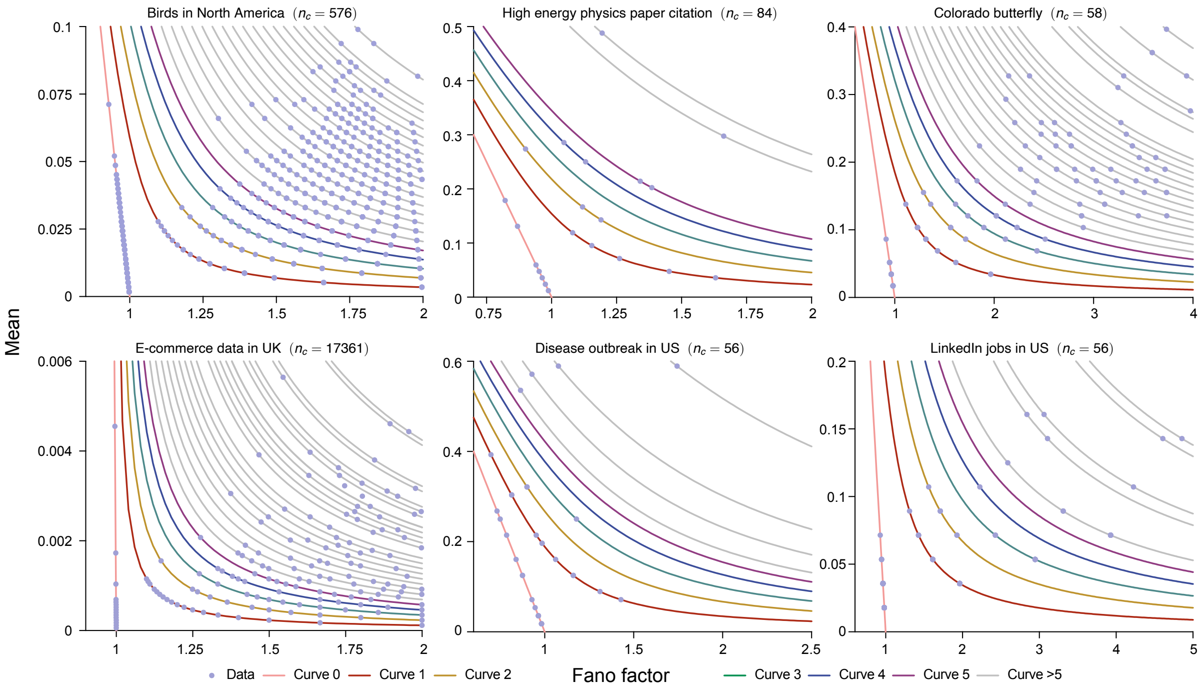

Figure S1: Mean-Fano factor plots of six different types of discrete and stochastic datasets which are not related to single-cell transcriptomics. The theoretical equation of Curve k is given by Eqs. (3)-(4) in the main text. The variable n_c is the number of columns in the count matrix. The number of points in the plot is equal to the number of rows in the count matrix. In all cases, the theoretical curves exactly pass through the points in the plot thus verifying the theory's accuracy for diverse types of datasets. Note that for visual clarity, we only plot those theoretical curves with $k > 5$ which explain the measured point positions.

References

[1] Hagemann-Jensen, M. *et al.* Single-cell RNA counting at allele and isoform resolution using Smart-seq3. *Nature Biotechnology* **38**, 708–714 (2020).

[2] Salmen, F. *et al.* High-throughput total RNA sequencing in single cells using VASA-seq. *Nature Biotechnology* **40**, 1780–1793 (2022).

[3] Saint, M. *et al.* Single-cell imaging and RNA sequencing reveal patterns of gene expression heterogeneity during fission yeast growth and adaptation. *Nature Microbiology* **4**, 480–491 (2019).

[4] Xia, C., Fan, J., Emanuel, G., Hao, J. & Zhuang, X. Spatial transcriptome profiling by MERFISH reveals subcellular RNA compartmentalization and cell cycle-dependent gene expression. *Proceedings of the National Academy of Sciences* **116**, 19490–19499 (2019).

[5] Hahaut, V. *et al.* Fast and highly sensitive full-length single-cell RNA sequencing using FLASH-seq. *Nature Biotechnology* **40**, 1447–1451 (2022).

[6] Bonter, D. N. & Greig, E. I. Over 30 years of standardized bird counts at supplementary feeding stations in north america: A citizen science data report for project feederwatch. *Frontiers in Ecology and Evolution* **9**, 619682 (2021).

[7] Oliver, J., Prudic, K. & Collinge, S. Boulder county open space butterfly diversity and abundance: Ecological archives e087-061. *Ecology* **87**, 1066–1066 (2006).

[8] Chen, D., Sain, S. L. & Guo, K. Data mining for the online retail industry: A case study of RFM model-based customer segmentation using data mining. *Journal of Database Marketing & Customer Strategy Management* **19**, 197–208 (2012).
